# Baseline CT radiomics for early prediction of post-traumatic hydrocephalus in traumatic brain injury: development and temporal validation

**DOI:** 10.3389/fneur.2026.1861928

**Published:** 2026-09-24

**Authors:** Chunwang Wu, Kaijie Yu, Xujian Shui, Xiaofeng Lin, Yuejun Fan, Yunxiang Chen

**Affiliations:** Department of Neurosurgery, Affiliated Jinhua Hospital, Zhejiang University School of Medicine, Jinhua, Zhejiang, China

**Keywords:** computed tomography, machine learning, post-traumatic hydrocephalus, radiomics, traumatic brain injury

## Abstract

**Background:**

Post-traumatic hydrocephalus (PTH) impairs recovery after traumatic brain injury (TBI), yet reliable early risk stratification based solely on baseline assessment remains limited. We aimed to develop and temporally validate a preoperative CT–based radiomics model for early prediction of PTH and to assess the incremental value of clinical predictors.

**Methods:**

In this retrospective single-center cohort, 341 consecutive adults undergoing neurosurgical treatment for TBI were included. Patients treated in 2021–2023 (*n* = 289) formed the training cohort, and those treated in 2024 (*n* = 52) served as a temporally independent validation cohort. A total of 11,998 radiomics features were extracted from 14 automatically segmented intracranial regions on the last preoperative CT. Feature selection was performed using mutual information filtering and LASSO regression. Machine learning classifiers were benchmarked, and a clinical–radiomics fusion model was constructed using stacking. Model performance was evaluated using discrimination, calibration, and decision curve analysis.

**Results:**

PTH occurred in 27.3% of the training cohort and 23.1% of the validation cohort. In temporal validation, the clinical, radiomics, and fusion models achieved AUCs of 0.769 (95% CI, 0.608–0.906), 0.783 (95% CI, 0.604–0.929), and 0.865 (95% CI, 0.735–0.958), respectively. The fusion model also demonstrated acceptable calibration and greater net benefit across clinically relevant thresholds.

**Conclusion:**

Baseline preoperative CT radiomics enables early risk stratification of PTH after TBI. Integration with clinical predictors provides incremental predictive value under temporal validation and may support individualized surveillance.

## 1 Introduction

Traumatic brain injury (TBI) represents a substantial global public health burden, with more than 20 million new cases reported annually worldwide (1). It contributes significantly to mortality and long-term disability and is frequently accompanied by a wide range of medical and neurological complications (2). Among these, post-traumatic hydrocephalus (PTH) is one of the most clinically significant yet potentially treatable sequelae, with reported incidence rates ranging from 8 to 36% (3), particularly in patients undergoing surgical intervention.

PTH often necessitates cerebrospinal fluid diversion procedures and has been associated with prolonged hospitalization, delayed neurological recovery, and an increased risk of long-term cognitive and functional impairment. The underlying pathophysiological mechanisms are multifactorial, involving hemorrhage- and subarachnoid hemorrhage–related inflammatory responses, impaired cerebrospinal fluid absorption, alterations in ventricular compliance, and microstructural injury to the periventricular white matter (4–7).

Current risk stratification for PTH largely relies on demographic factors, admission neurological status, and qualitative radiological features such as subarachnoid hemorrhage severity, intraventricular hemorrhage, and ventricular morphology (8, 9). Although these variables are clinically meaningful, traditional models often demonstrate only moderate discrimination and are limited by heterogeneous patient populations and interobserver variability in imaging interpretation. Furthermore, many previous studies incorporate postoperative clinical or imaging variables (10), thereby limiting their ability to provide true early risk stratification based on baseline assessment. The lack of prediction tools derived exclusively from baseline imaging limits early risk stratification and individualized surveillance planning.

Radiomics enables high-throughput extraction of quantitative imaging features that capture intensity distribution, spatial texture, and structural heterogeneity beyond visual interpretation (11). CT radiomics has demonstrated predictive value in neurotrauma and hemorrhage-related complications, including outcome prognostication and secondary injury prediction (12, 13). These findings suggest that routinely acquired baseline CT images may contain latent quantitative signatures reflecting hemorrhage distribution, microenvironmental alteration, and early CSF circulation disturbance that are not fully captured by conventional grading systems.

Accordingly, we aimed to develop and temporally validate a baseline preoperative CT–based radiomics model for early prediction of PTH in patients with TBI. We further evaluated whether integration with clinical predictors provides incremental predictive value and whether model performance remains stable in a chronologically independent validation cohort.

## 2 Materials and methods

### 2.1 Study design and ethical approval

This retrospective, single-center study was conducted at Affiliated Jinhua Hospital, Zhejiang University School of Medicine. The study protocol was approved by the Ethics Committee of Affiliated Jinhua Hospital, Zhejiang University School of Medicine (Approval No. 2026-69; approved on 28 February 2026), and the requirement for written informed consent was waived due to the retrospective design.

### 2.2 Study population

Consecutive adult patients with TBI who underwent neurosurgical treatment between January 2021 and June 2024 were retrospectively reviewed. Inclusion criteria were: (1) confirmed history of TBI; (2) age ≥ 18 years; (3) surgical treatment performed at our institution; (4) survival for at least 6 months after TBI; and (5) complete clinical and imaging data. Exclusion criteria were: (1) death or loss to follow-up; (2) age < 18 years; (3) pre-existing hydrocephalus, brain atrophy, or other neurological diseases before TBI; (4) prior craniotomy; and (5) follow-up duration < 6 months.

### 2.3 Outcome definition

PTH was defined as postoperative hydrocephalus occurring within 6 months after TBI surgery and fulfilling both radiological and clinical criteria. Radiological diagnosis required new or progressive ventricular enlargement on follow-up CT (Evans index > 0.30, temporal horn ≥ 2 mm, rounded third ventricle, or disproportionate ventricular enlargement). Clinical diagnosis required new neurological deterioration not attributable to other causes (14, 15). All cases were independently adjudicated by two blinded neurosurgeons.

### 2.4 Dataset splitting

Patients treated between January 2021 and December 2023 (*n* = 289) were assigned to the training cohort, and patients treated between January 2024 and June 2024 (*n* = 52) were assigned to the temporal validation cohort (Figure 1). The temporal validation cohort was exclusively used for independent validation and was not involved in model development.

**Figure 1:**
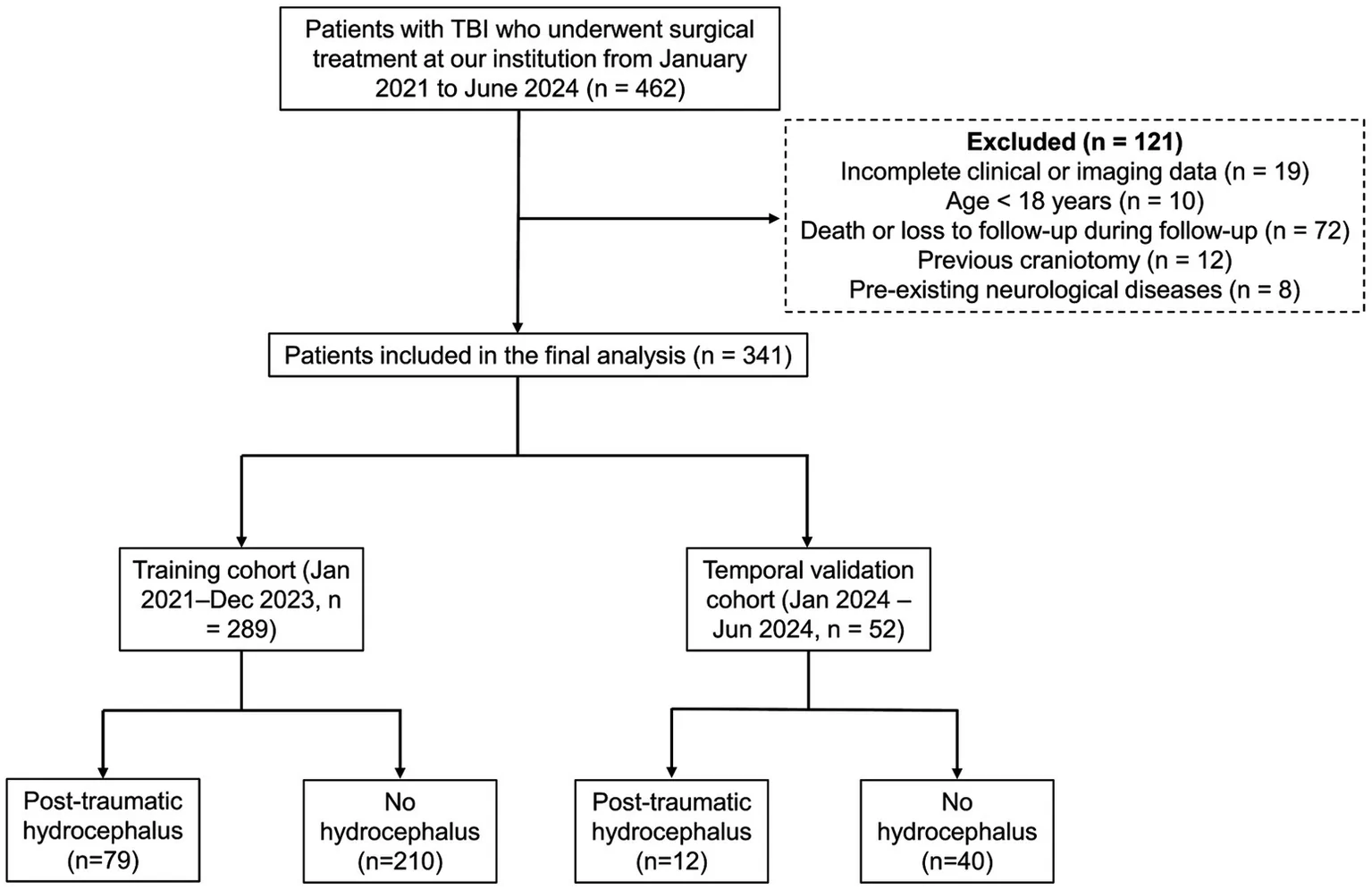
Recruitment pathway for patients in this study.

### 2.5 Clinical variables and routine imaging parameters

Clinical variables, including admission neurological status (e.g., Glasgow Coma Scale score) and routine radiological parameters (e.g., modified Fisher grade and presence of skull fracture), were systematically extracted from the institutional electronic medical record system and the hospital Picture Archiving and Communication System (PACS) based on standardized clinical assessment and imaging review protocols.

Variables with missing values ≥20% were excluded from further analysis. For variables with missing rates < 20%, missing values were handled using median imputation during model development.

### 2.6 CT acquisition

All patients underwent non-contrast cranial CT using a 64-slice spiral CT scanner (uCT 710, United Imaging Healthcare, Shanghai, China) under a standardized institutional protocol. Axial images were acquired from the skull base to the vertex. When clinically required, spinal immobilization was maintained during CT using a spinal board positioned below the neck and a rigid plastic cervical collar, both located predominantly outside the intracranial acquisition range. The tube voltage was set to 120 kV with automatic tube current modulation. Images were reconstructed with a slice thickness of 1 mm without interslice gap. The last CT examination obtained before surgery was selected for radiomics analysis because it represented the most recent imaging information available at the time of surgical decision-making. The interval between trauma and the selected CT examination varied according to the patient’s clinical condition and emergency management requirements.

### 2.7 Image quality assessment

Before automated segmentation and radiomics feature extraction, all candidate CT examinations were independently reviewed by two neurosurgeons experienced in neuroimaging, who were blinded to the PTH outcome. The original axial images and, when necessary, coronal and sagittal reconstructions were assessed for completeness of intracranial coverage and the presence of motion, beam-hardening, streak, or device-related artifacts. Examinations were considered unsuitable for radiomics analysis when image degradation obscured the target intracranial structures or prevented reliable segmentation. Such examinations were classified under the exclusion category of incomplete clinical or imaging data. Disagreements regarding image eligibility were resolved by consensus.

### 2.8 ROI segmentation and radiomics feature extraction

Regions of interest (ROIs) were automatically segmented using the MONAI Auto3DSeg module (Brain and Intracranial Hemorrhage model) implemented in 3D Slicer (version 5.10.0; https://www.slicer.org). Fourteen anatomical and hemorrhagic ROIs were analyzed per patient (Supplementary Table S1). All automatically generated ROI masks were visually reviewed against the corresponding CT images before radiomics feature extraction. Radiomics feature extraction was performed using the SlicerRadiomics extension (PyRadiomics backend) within the same 3D Slicer environment, following the Image Biomarker Standardization Initiative (IBSI) guidelines. For each ROI, 857 quantitative features were extracted, including shape features, first-order statistics, and texture features derived from gray-level co-occurrence matrix (GLCM), gray-level run-length matrix (GLRLM), gray-level size zone matrix (GLSZM), gray-level dependence matrix (GLDM), and neighboring gray-tone difference matrix (NGTDM). Wavelet-transformed images were additionally used to generate high-dimensional texture features. In total, 11,998 radiomics features were generated per patient prior to feature selection. Detailed radiomics extraction parameters and Image Biomarker Standardisation Initiative (IBSI) compliance statements are provided in Supplementary methods.

### 2.9 Feature selection and radiomics model development

Feature selection and radiomics model development were conducted exclusively in the training cohort. Radiomics features were standardized using Z-score normalization based on the training set mean and standard deviation, and the same parameters were applied to the temporal validation cohort.

Given the high dimensionality, a two-step feature selection strategy was applied. First, mutual information analysis ranked features according to relevance to postoperative hydrocephalus, and top-ranked features were retained. Second, least absolute shrinkage and selection operator (LASSO) regression with 5-fold cross-validation was performed for further dimensionality reduction. The optimal regularization parameter (λ) was selected based on the minimum cross-validated binomial deviance, and features with non-zero coefficients were retained.

Using the selected features, eight machine learning classifiers were trained, including logistic regression, elastic net, support vector machine with radial basis function kernel, random forest, extra trees, gradient boosting decision tree, histogram-based gradient boosting, and k-nearest neighbors. Performance in the training cohort was evaluated using 5-fold cross-validation to generate out-of-fold (OOF) predictions. The model achieving the highest cross-validated area under the receiver operating characteristic curve (AUC) was selected as the optimal radiomics model.

Feature-selection stability was assessed exclusively in the training cohort using 50 repetitions of stratified five-fold cross-validation, yielding 250 training subsets. Within each subset, mutual-information filtering and L1-penalized logistic regression were repeated. The selection frequency of each candidate feature was calculated as the proportion of subsets in which it was retained. The temporal validation cohort was not used in the stability analysis.

### 2.10 Clinical model development

A clinical prediction model was developed using logistic regression in the training cohort. Candidate clinical variables and routine CT imaging parameters were first assessed using univariate logistic regression. Variables with *p* < 0.050 were subsequently entered into a multivariable logistic regression model to identify independent predictors. Adjusted odds ratios (ORs) with 95% confidence intervals (CIs) were reported. The final clinical model was fitted on the training cohort and evaluated in the temporal validation cohort.

### 2.11 Fusion model development

A clinical–radiomics fusion model was constructed using a stacking strategy. Briefly, predicted probabilities from the clinical model and the optimal radiomics model were combined as inputs to a logistic regression meta-learner. The meta-learner was trained using the training cohort OOF predictions to reduce overfitting, and then applied to the temporal validation cohort for independent validation.

### 2.12 Model evaluation

Model discrimination was assessed using the area under the receiver operating characteristic curve (AUC) and average precision (AP). Calibration was evaluated using calibration curves, Brier scores, and the Hosmer–Lemeshow goodness-of-fit test. Calibration curves were generated using out-of-fold predictions in the training cohort and predicted probabilities in the temporal validation cohort. Clinical usefulness was assessed using decision curve analysis (DCA) (Figure 2).

**Figure 2:**
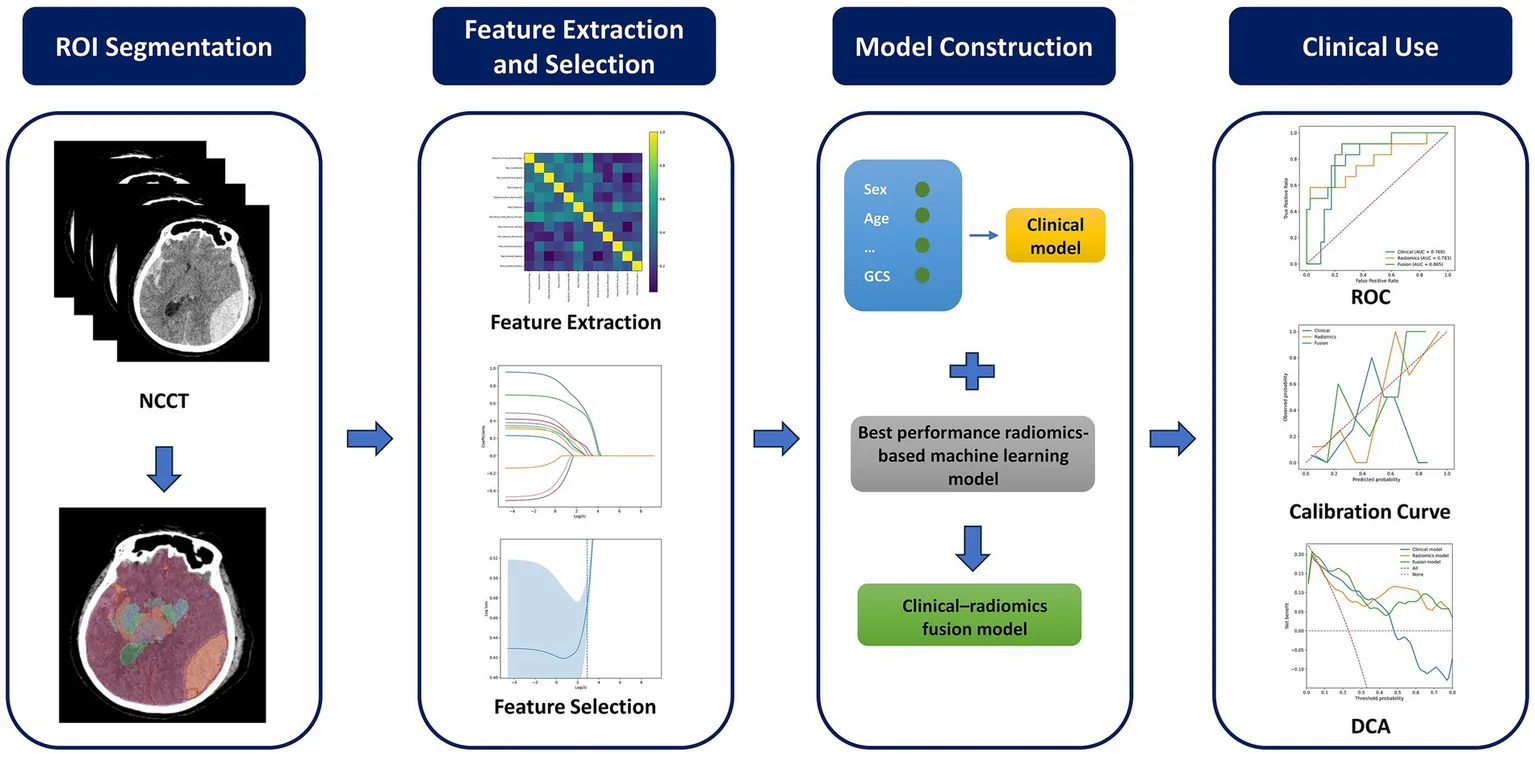
The flowchart of our study.

### 2.13 Statistical analysis

Continuous variables were expressed as mean ± standard deviation or median (interquartile range), as appropriate, and categorical variables were presented as counts and percentages. Between-group comparisons were performed using the independent-samples *t* test or Mann–Whitney U test for continuous variables and the χ^2^ test or Fisher’s exact test for categorical variables. No synthetic oversampling, undersampling, or class weighting was applied. Stratified five-fold cross-validation was used to preserve the outcome distribution within each fold. Ninety-five percent confidence intervals for model performance metrics were estimated using the percentile method based on 5,000 stratified patient-level bootstrap resamples. Model-specific classification thresholds were determined from the training-cohort out-of-fold predictions using the Youden index and were applied unchanged to the temporal validation cohort. Pairwise comparisons of AUCs were performed using the DeLong test. All statistical tests were two-sided, and a *p* value <0.05 was considered statistically significant. All statistical analyses and machine learning modeling were conducted using Python (version 3.13.9) with scikit-learn (version 1.7.2) and R software (version 4.3.1).

## 3 Results

### 3.1 Patient characteristics

A total of 462 patients with TBI were screened between January 2021 and June 2024. After applying the inclusion and exclusion criteria, 341 patients were included in the final analysis. The training cohort comprised 289 patients treated between January 2021 and December 2023, and the temporal validation cohort comprised 52 patients treated between January 2024 and June 2024.

In the training cohort, PTH occurred in 79/289 patients (27.3%), and in the temporal validation cohort, PTH occurred in 12/52 patients (23.1%). Baseline characteristics are summarized in Table 1.

**Table 1:** Baseline characteristics of the study population.

Variable	Training cohort (2021–2023, *n* = 289)	Temporal validation cohort (2024, *n* = 52)
Age, years	58.4 ± 14.5	59.2 ± 15.1
GCS score on admission	7.5 ± 3.4	7.8 ± 3.8
Modified Fisher score	1.5 ± 1.1	1.6 ± 1.1
Sex		
Female	79 (27.3%)	14 (26.9%)
Male	210 (72.7%)	38 (73.1%)
Hypertension		
No	235 (81.3%)	43 (82.7%)
Yes	54 (18.7%)	9 (17.3%)
Diabetes mellitus		
No	271 (93.8%)	49 (94.2%)
Yes	18 (6.2%)	3 (5.8%)
Smoking history		
No	231 (79.9%)	44 (84.6%)
Yes	58 (20.1%)	8 (15.4%)
Alcohol consumption		
No	236 (81.7%)	42 (80.8%)
Yes	53 (18.3%)	10 (19.2%)
Pupillary response		
Reactive	41 (14.2%)	8 (15.4%)
Sluggish	135 (46.7%)	29 (55.8%)
Non-reactive	113 (39.1%)	15 (28.8%)
Midline shift >5 mm		
No	107 (37.0%)	22 (42.3%)
Yes	182 (63.0%)	30 (57.7%)
Skull fracture		
None	242 (83.7%)	45 (86.5%)
Linear	19 (6.6%)	1 (1.9%)
Depressed	28 (9.7%)	6 (11.5%)

Continuous variables are presented as mean ± standard deviation, and categorical variables are presented as number (percentage). GCS, Glasgow Coma Scale.

### 3.2 Clinical risk factor analysis and clinical model development

Univariate and multivariable logistic regression analyses were performed to identify independent clinical predictors of PTH (Table 2). In multivariable analysis, age, admission Glasgow Coma Scale (GCS) score, and modified Fisher grade remained independently associated with PTH.

**Table 2:** Univariable and multivariable logistic regression analyses.

Variable	Univariable OR (95% CI)	*p* value	Multivariable OR (95% CI)	*p* value
Age (per year)	1.07 (1.04–1.09)	<0.001	1.05 (1.03–1.08)	<0.001
Male sex	1.06 (0.62–1.83)	0.822	–	–
Hypertension	1.02 (0.55–1.89)	0.953	–	–
Diabetes mellitus	1.76 (0.70–4.39)	0.228	–	–
Smoking history	0.77 (0.41–1.45)	0.419	–	–
Alcohol consumption	1.36 (0.75–2.46)	0.316	–	–
GCS score (per point)	0.72 (0.64–0.80)	<0.001	0.74 (0.66–0.83)	<0.001
Modified Fisher score (per grade)	1.92 (1.55–2.38)	<0.001	1.71 (1.34–2.19)	<0.001
Pupillary response (ordinal)	3.01 (1.98–4.58)	<0.001	–	–
Midline shift >5 mm	2.18 (1.27–3.72)	0.004	–	–
Skull fracture (ordinal)	0.80 (0.52–1.21)	0.286	–	–

Odds ratios (ORs) are presented with 95% confidence intervals (CIs). Continuous variables were analyzed per unit increase. Pupillary response and skull fracture were treated as ordinal variables. “–” Indicates that the variable was not included in the multivariable model.

Specifically, older age was associated with an increased risk (OR 1.05 (1.03–1.08), *p* < 0.001), whereas higher GCS was protective (OR 0.74 (0.66–0.83), *p* < 0.001). Higher modified Fisher grade was associated with increased risk (OR 1.71 (1.34–2.19), *p* < 0.001).

The clinical model constructed using these predictors demonstrated good discrimination, with an AUC of 0.835 (95% CI, 0.785–0.880) in the training cohort (out-of-fold predictions) and 0.769 (95% CI, 0.608–0.906) in the temporal validation cohort. The Hosmer–Lemeshow test suggested acceptable calibration in the training cohort (χ^2^ = 7.54, df = 8, *p* = 0.479) and borderline calibration in the temporal validation cohort (χ^2^ = 15.26, df = 8, *p* = 0.054).

### 3.3 Radiomics feature selection and radiomics model performance

Fourteen ROIs were segmented per patient. For each ROI, 857 radiomics features were extracted, resulting in 11,998 features per patient before feature selection. To reduce dimensionality, mutual information filtering retained the top 50 features, followed by LASSO regression, which selected 12 features with non-zero coefficients to construct the radiomics signature (Figure 3; Supplementary Table S2). Feature-selection stability was assessed using 50 repetitions of stratified five-fold cross-validation in the training cohort. Among the 12 features included in the final radiomics signature, the median selection frequency was 99.4% (range, 75.6–100.0%), and 11 of 12 features were retained in at least 80% of the resampled subsets (Supplementary Table S3; Supplementary Figure S1).

**Figure 3:**
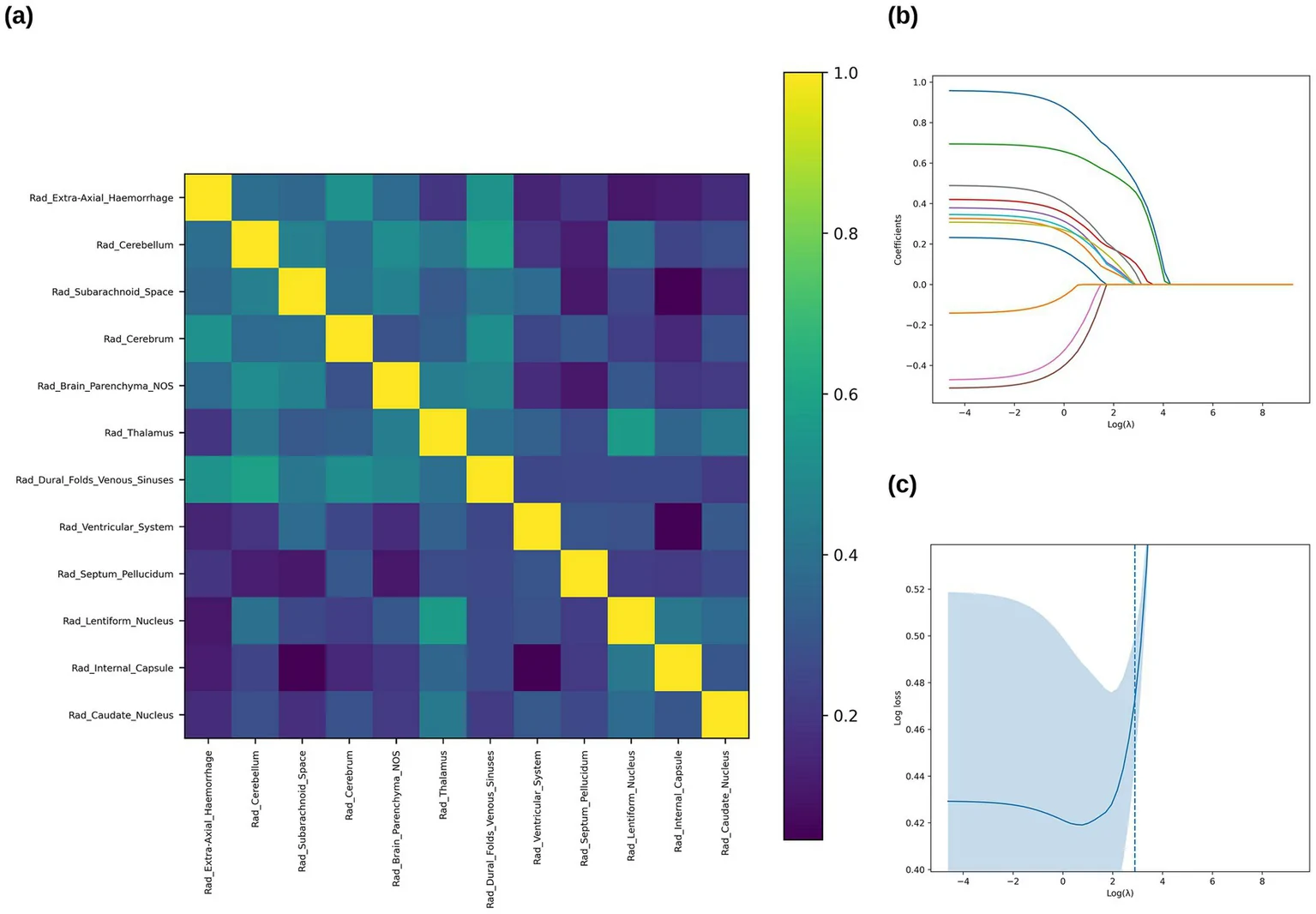
Radiomics feature correlation and LASSO-based feature selection in the training cohort. **(a)** Correlation heatmap showing pairwise Pearson correlation coefficients among representative radiomics features extracted from different anatomical ROIs. Color intensity reflects the magnitude of correlation (r), with the diagonal indicating self-correlation (*r* = 1). **(b)** LASSO coefficient profiles plotted against log(λ). Each curve represents an individual radiomics feature, demonstrating progressive coefficient shrinkage as the regularization strength increases. **(c)** Five-fold cross-validation curve for tuning parameter selection in the LASSO model. The solid line represents the mean binomial deviance across folds, and the shaded region indicates ±1 standard error. The vertical dashed line denotes the optimal λ corresponding to the minimum cross-validated deviance, and features with non-zero coefficients at this λ were retained for subsequent model construction.

Eight machine learning classifiers were evaluated (Table 3). The logistic regression classifier achieved the highest AUC in the temporal validation cohort (AUC, 0.783; 95% CI, 0.604–0.929) and was therefore selected as the optimal radiomics model. The next-best model was ElasticNet (AUC, 0.781; 95% CI, 0.598–0.929).

**Table 3:** Performance of radiomics-based machine learning models.

Model	Train OOF AUC (95% CI)	Train OOF AP	Temporal test AUC (95% CI)	Temporal test AP	Temporal test Brier
Logistic regression	0.841 (0.783–0.889)	0.713	**0.783 (0.604–0.929)**	0.705	0.122
Elastic net	0.841 (0.786–0.890)	0.714	0.781 (0.598–0.929)	0.704	0.121
SVM (RBF)	0.802 (0.741–0.860)	0.652	0.754 (0.569–0.921)	0.698	0.129
Extra trees	0.855 (0.806–0.901)	0.71	0.754 (0.565–0.915)	0.675	0.146
Random forest	0.858 (0.806–0.905)	0.708	0.736 (0.545–0.900)	0.635	0.15
HistGradientBoosting	0.859 (0.810–0.905)	0.707	0.721 (0.517–0.900)	0.58	0.169
KNN	0.821 (0.763–0.875)	0.666	0.703 (0.485–0.894)	0.659	0.153
GBDT	0.846 (0.793–0.896)	0.723	0.679 (0.471–0.863)	0.52	0.195

AUC, area under the ROC curve; AP, average precision (PR-AUC); Brier, Brier score. Bold indicates the best model by temporal test AUC (primary selection criterion). Elastic net achieved the lowest Brier score, with similar discrimination to logistic regression.

The optimal radiomics model achieved an AUC of 0.841 (95% CI, 0.783–0.889) in the training cohort (out-of-fold) and 0.783 (95% CI, 0.604–0.929) in the temporal validation cohort. The corresponding average precision (AP) was 0.713 in the training cohort and 0.705 in the temporal validation cohort, with Brier scores of 0.133 and 0.122, respectively. The Hosmer–Lemeshow test indicated χ^2^ = 17.87, df = 8, *p* = 0.022 in the training cohort and χ^2^ = 9.97, df = 8, *p* = 0.267 in the temporal validation cohort.

### 3.4 Clinical–radiomics fusion model

A fusion model was developed using a stacking strategy that combined the predicted probabilities from the clinical model and the optimal radiomics model as inputs to a logistic regression meta-learner.

The fusion model demonstrated improved discrimination compared with the individual models. In the training cohort, the fusion model achieved an AUC of 0.894 (95% CI, 0.851–0.932), compared with 0.835 (95% CI,0.785–0.880) for the clinical model and 0.841 (95% CI, 0.783–0.889) for the radiomics model (Figure 4a). In the temporal validation cohort, the fusion model achieved an AUC of 0.865 (95% CI, 0.735–0.958), compared with 0.769 (95% CI, 0.608–0.906) and 0.783 (95% CI, 0.604–0.929) for the clinical and radiomics models, respectively (Figure 4b).

**Figure 4:**
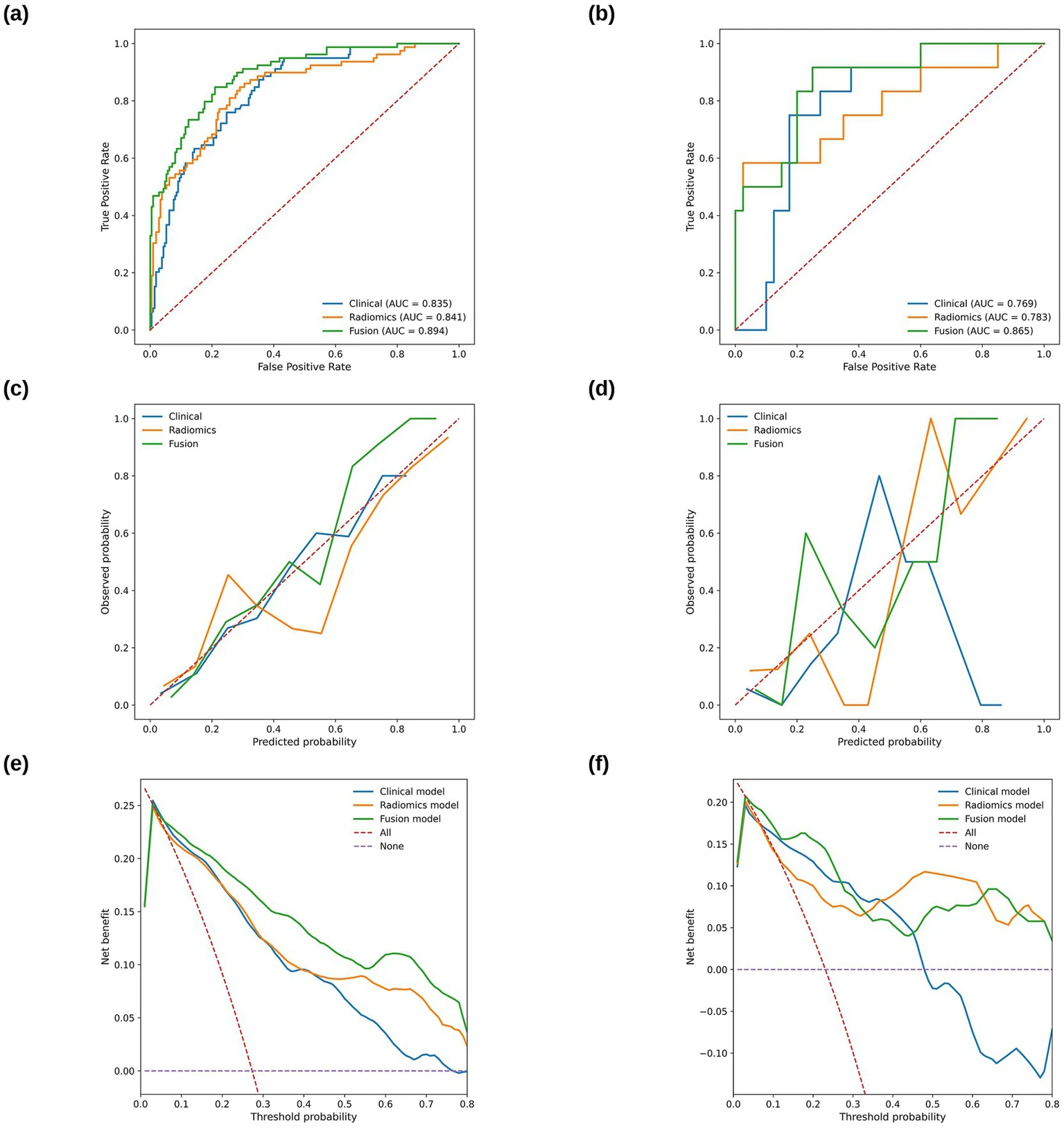
Performance evaluation of the clinical, radiomics, and fusion models in the training and temporal validation cohorts. **(a)** Receiver operating characteristic (ROC) curves in the training cohort. **(b)** ROC curves in the temporal validation cohort. **(c)** Calibration curves in the training cohort. **(d)** Calibration curves in the temporal validation cohort. **(e)** Decision curve analysis (DCA) in the training cohort. **(f)** DCA in the temporal validation cohort.

DeLong tests showed that the fusion model significantly outperformed the clinical model in the training cohort (*p* = 0.003) and in the temporal validation cohort (*p* = 0.039). The fusion model also outperformed the radiomics model in the training cohort (*p* = 0.002), while the difference was not statistically significant in the temporal validation cohort (*p* = 0.289).

At the classification thresholds determined from the training-cohort out-of-fold predictions, the fusion model achieved a sensitivity of 0.833, specificity of 0.800, accuracy of 0.808, and F1 score of 0.667 in the temporal validation cohort. The fusion model also achieved the highest average precision (0.736) among the three models. Complete validation-cohort performance metrics with 95% confidence intervals are presented in Table 4.

**Table 4:** Performance of the clinical, radiomics, and fusion models in the temporal validation cohort.

Metric	Clinical model (95% CI)	Radiomics model (95% CI)	Fusion model (95% CI)
AUC	0.769 (0.608–0.906)	0.783 (0.604–0.929)	**0.865 (0.735–0.958)**
Average precision	0.423 (0.317–0.684)	0.705 (0.495–0.900)	**0.736 (0.545–0.904)**
Brier score	0.157 (0.109–0.212)	0.122 (0.069–0.176)	**0.116 (0.077–0.159)**
Sensitivity	**0.833 (0.583–1.000)**	0.667 (0.417–0.917)	**0.833 (0.583–1.000)**
Specificity	0.625 (0.475–0.775)	0.700 (0.550–0.826)	**0.800 (0.675–0.900)**
Positive predictive value	0.400 (0.296–0.526)	0.400 (0.261–0.563)	**0.556 (0.400–0.750)**
Negative predictive value	0.926 (0.833–1.000)	0.875 (0.784–0.967)	**0.941 (0.865–1.000)**
Accuracy	0.673 (0.538–0.788)	0.692 (0.558–0.808)	**0.808 (0.692–0.904)**
F1 score	0.541 (0.410–0.667)	0.500 (0.313–0.667)	**0.667 (0.500–0.815)**

AUC, area under the ROC curve; CI, confidence interval; Bold indicates the best value for each metric.

Calibration assessment demonstrated acceptable agreement between predicted and observed risks for the fusion model in both cohorts (Figures 4c,d; Hosmer–Lemeshow: training χ^2^ = 9.33, df = 8, *p* = 0.315, temporal validation χ^2^ = 9.45, df = 8, *p* = 0.306).

### 3.5 Calibration and clinical utility

Calibration curves for the clinical, radiomics, and fusion models are shown in Figures 4c,d. Decision curve analysis demonstrated that the fusion model provided a higher net benefit across a wide range of threshold probabilities compared with the clinical and radiomics models in both the training and temporal validation cohorts (Figures 4e,f).

### 3.6 Contribution of clinical and radiomics components in the fusion model

The contribution of the clinical and radiomics predictions to the fusion model is illustrated in Figure 5. Both components contributed independently to the final fusion prediction, supporting the complementary value of integrating clinical information with radiomics signatures.

**Figure 5:**
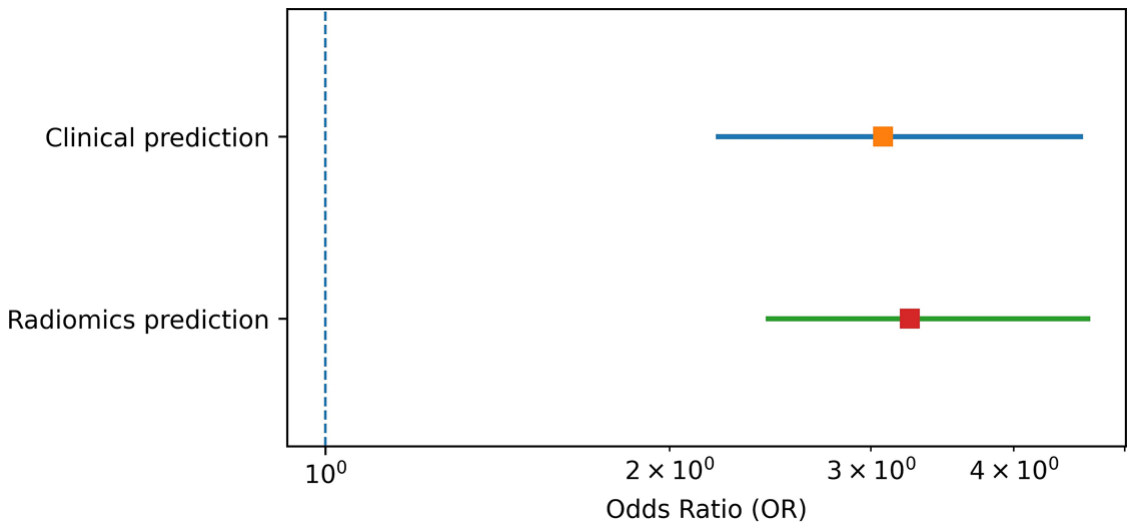
Odds ratios (ORs) for clinical and radiomics predictions. Forest plot showing the estimated odds ratios with corresponding 95% confidence intervals for the clinical prediction model and the radiomics prediction model. The vertical dashed line indicates the reference value (OR = 1).

Across models, performance differences between the training (out-of-fold) and temporal validation cohorts suggested no substantial evidence of overfitting, with consistent discrimination observed in the temporal validation cohort.

## 4 Discussion

In this retrospective study with chronological validation, we demonstrated that routinely acquired baseline preoperative CT contains reproducible quantitative signatures associated with subsequent PTH development. A radiomics signature derived exclusively from the last preoperative scan maintained discriminatory capacity in a temporally independent cohort, supporting its robustness beyond internal resampling. Integration of radiomics-derived probabilities with established clinical predictors further improved model performance, highlighting the complementary contributions of imaging heterogeneity and conventional severity indicators. Because the entire framework was restricted to baseline assessment, the model enables early risk stratification at a clinically actionable stage, without reliance on postoperative evolution or follow-up findings.

Advanced age was independently associated with increased risk of PTH. Aging has been linked to reduced brain compliance, altered ventricular elasticity, and impaired cerebrospinal fluid (CSF) absorption capacity (16). Lower admission GCS score also emerged as an independent predictor (17). Severe primary brain injury may reflect greater tissue disruption, inflammatory response, and subarachnoid blood burden, all of which may impair CSF circulation. Modified Fisher grade was strongly associated with PTH risk, consistent with prior evidence demonstrating that subarachnoid hemorrhage and intraventricular blood contribute to arachnoid granulation obstruction and inflammatory fibrosis (2). These findings align with previous clinical investigations identifying hemorrhage burden and neurological severity as major determinants of post-traumatic ventricular enlargement (18, 19). However, the discrimination of the clinical model alone was moderate in temporal validation (AUC, 0.769; 95% CI, 0.608–0.906), suggesting that conventional predictors may not fully capture the imaging heterogeneity underlying CSF circulation disturbances.

Radiomics enables high-throughput extraction of quantitative features describing intensity distribution, spatial texture, and structural complexity beyond visual assessment (20, 21). In neuroimaging, CT-based radiomics has demonstrated predictive value for hemorrhage expansion, outcome stratification, and secondary injury progression (22–24). The present radiomics model achieved stable discrimination across training and temporal cohorts, suggesting that baseline CT contains reproducible quantitative signatures associated with subsequent PTH development.

From a mechanistic perspective, radiomics features may encode subtle patterns of hemorrhage distribution, periventricular edema heterogeneity, and microstructural disruption that precede overt ventricular enlargement. Experimental and imaging studies have shown that blood degradation products and inflammatory cascades alter periventricular white matter integrity and CSF flow dynamics (2). Such microenvironmental alterations are unlikely to be fully captured by qualitative grading systems but may be reflected in texture-based radiomics features. Importantly, predictors were extracted exclusively from baseline CT, preserving temporal causality. Many prior prediction models incorporate postoperative or follow-up variables, thereby limiting early risk stratification utility.

The fusion model outperformed both individual models in the temporal validation cohort, achieving an AUC of 0.865 (95% CI, 0.735–0.958), compared with 0.769 (95% CI, 0.608–0.906) for the clinical model and 0.783 (95% CI, 0.604–0.929) for the radiomics model. This finding suggests that radiomics provides non-redundant information complementary to clinical variables. Multimodal integration has been increasingly recognized as a strategy to enhance predictive performance in radiology (25). Clinical variables capture global injury severity, whereas radiomics features quantify localized structural heterogeneity. The stacking framework used in this study allowed probabilistic integration while reducing overfitting risk by leveraging out-of-fold predictions. The absence of substantial performance degradation in temporal validation further supports model robustness.

Previous studies on PTH prediction have predominantly relied on demographic variables, hemorrhage grading scales, and postoperative clinical evolution (26, 27). Few investigations have explored quantitative radiomics approaches in this context, and even fewer have employed temporal validation. Temporal validation is increasingly emphasized as a critical step to assess model transportability and mitigate optimism bias. By adopting a chronological split, the present study more closely simulates real-world prospective deployment conditions.

Early identification of patients at high risk of PTH may facilitate closer neurological monitoring, earlier or more frequent follow-up CT, and earlier outpatient neurosurgical assessment. Delayed recognition of hydrocephalus has been associated with worse neurological recovery and prolonged rehabilitation (2). Because non-contrast CT is routinely acquired in acute TBI management, the proposed model would not require additional imaging. In a future clinical workflow, CT images could be transferred directly from the PACS for automated image-quality assessment, intracranial segmentation, radiomics feature extraction, and fusion-model risk calculation. Cases with inadequate image quality or uncertain segmentation could be flagged for manual review, and the resulting risk estimate could be returned to the PACS or electronic medical record as a clinical decision-support output. Patients identified as being at high risk could receive an individualized surveillance plan. If progressive ventricular enlargement and compatible neurological deterioration subsequently occur, these patients could undergo earlier evaluation of CSF circulation and the potential need for temporary or permanent CSF diversion. Importantly, the model should be regarded as a risk-stratification tool rather than a replacement for clinical judgment or a direct indication for CSF diversion. Prospective clinical evaluation, workflow automation, and predefined risk thresholds are required before routine implementation.

Several limitations should be acknowledged. First, this was a retrospective single-center study, and both cohorts shared the same scanner platform and acquisition protocol. Although temporal validation provides a more rigorous assessment than random internal splitting, it does not constitute true external validation. Radiomics features may be sensitive to scanner characteristics and acquisition and reconstruction parameters; therefore, multicenter external validation across different institutions, scanners, and imaging protocols is required before clinical implementation. Second, automated segmentation improves scalability but may introduce boundary errors; robustness analyses against segmentation variability remain important. Third, although immobilization devices were located predominantly outside the intracranial acquisition range, subtle effects on image noise and radiomics features cannot be completely excluded. Fourth, the interval between trauma and the selected preoperative CT was not uniform, and the temporal evolution of traumatic intracranial abnormalities may have introduced variability in the extracted radiomics features. Fifth, the temporal validation cohort was relatively small and included only 12 PTH events, resulting in relatively wide confidence intervals and limited precision of the performance estimates. Finally, radiomics features are mathematical descriptors and do not directly establish mechanistic causality; future studies integrating longitudinal imaging, CSF flow metrics, or biological correlates may improve interpretability.

## 5 Conclusion

Preoperative CT radiomics enables prediction of PTH in TBI and maintains discrimination under temporal validation. Integration with clinical predictors provides incremental value and may facilitate individualized perioperative risk stratification.

## Data Availability

The original contributions presented in the study are included in the article/Supplementary material, further inquiries can be directed to the corresponding author.
