# Supplementary material for: Baseline CT radiomics for early prediction of post-traumatic hydrocephalus in traumatic brain injury: development and temporal validation
Source: Front Neurol. 2026 Sep 24;17:1861928. doi: 10.3389/fneur.2026.1861928 (PMC13646725; doi:10.3389/fneur.2026.1861928)
Supplement: Supplementary file 1 [file Supplementary_file_1.DOCX]

**Supplementary Methods. Radiomics extraction settings and IBSI compliance statement**

Radiomics feature extraction was performed using the SlicerRadiomics extension (PyRadiomics backend) within 3D Slicer (version 5.10.0). Feature definitions follow the Image Biomarker Standardisation Initiative (IBSI).

Feature classes enabled: shape, shape2D, first-order statistics, GLCM, GLRLM, GLSZM, GLDM, and NGTDM.

Image filters: wavelet features were enabled; Laplacian of Gaussian (LoG) filters were not used.

Discretization: fixed bin width = 25 (grayscale discretization).

GLCM setting: symmetric GLCM was enforced.

Resampling: No additional voxel resampling was applied in SlicerRadiomics. Radiomics features were computed based on the original reconstructed CT resolution (1 mm slice thickness).

Quality control: the same institutional CT acquisition protocol and reconstruction settings were used across the study period. All ROIs were generated automatically using MONAI Auto3DSeg and visually checked for gross failures prior to feature extraction.

Reproducibility: software versions for model development were reported in the main Methods section; all feature extraction settings listed above were kept constant for all patients.
