# Supplementary material for: Baseline CT radiomics for early prediction of post-traumatic hydrocephalus in traumatic brain injury: development and temporal validation
Source: Front Neurol. 2026 Sep 24;17:1861928. doi: 10.3389/fneur.2026.1861928 (PMC13646725; doi:10.3389/fneur.2026.1861928)
Supplement: Supplementary file 2 [file Table_1.DOCX]

# Supplementary Table S1. Segmented Regions of Interest (ROIs)

| No. | Region of Interest |
| --- | --- |
| 1 | Brain Parenchyma NOS |
| 2 | Subarachnoid Space |
| 3 | Dural Folds and Venous Sinuses |
| 4 | Septum Pellucidum |
| 5 | Cerebellum |
| 6 | Caudate Nucleus |
| 7 | Lentiform Nucleus |
| 8 | Insular Cortex |
| 9 | Internal Capsule |
| 10 | Ventricular System |
| 11 | Cerebrum |
| 12 | Thalamus |
| 13 | Intracerebral Hemorrhage |
| 14 | Extra-Axial Hemorrhage |
