# Supplementary material for: Baseline CT radiomics for early prediction of post-traumatic hydrocephalus in traumatic brain injury: development and temporal validation
Source: Front Neurol. 2026 Sep 24;17:1861928. doi: 10.3389/fneur.2026.1861928 (PMC13646725; doi:10.3389/fneur.2026.1861928)
Supplement: Supplementary file 3 [file Table_2.DOCX]

# Supplementary Table S2. Final Radiomics Features Included in Model Development

Radiomics features were selected using mutual information filtering followed by LASSO regression in the training cohort. The table below reports the final non-zero coefficient features used to construct the radiomics signature.

SelectedFeatures

Rad_Extra-Axial_Haemorrhage

Rad_Cerebellum

Rad_Subarachnoid_Space

Rad_Cerebrum

Rad_Brain_Parenchyma_NOS

Rad_Thalamus

Rad_Dural_Folds_Venous_Sinuses

Rad_Ventricular_System

Rad_Septum_Pellucidum

Rad_Lentiform_Nucleus

Rad_Internal_Capsule

Rad_Caudate_Nucleus
