# Supplementary material for: Baseline CT radiomics for early prediction of post-traumatic hydrocephalus in traumatic brain injury: development and temporal validation
Source: Front Neurol. 2026 Sep 24;17:1861928. doi: 10.3389/fneur.2026.1861928 (PMC13646725; doi:10.3389/fneur.2026.1861928)
Supplement: Supplementary file 4 [file Table_3.DOCX]

# Supplementary Table S3. Feature Selection Stability Analysis

Feature stability was evaluated using repeated cross-validation in the training cohort. Selection frequency represents the proportion of times each feature was retained during LASSO selection.

| feature | selected_count | selected_rate |
| --- | --- | --- |
| Rad_Brain_Parenchyma_NOS | 250 | 1.0 |
| Rad_Cerebrum | 250 | 1.0 |
| Rad_Extra-Axial_Haemorrhage | 250 | 1.0 |
| Rad_Ventricular_System | 250 | 1.0 |
| Rad_Subarachnoid_Space | 250 | 1.0 |
| Rad_Dural_Folds_Venous_Sinuses | 249 | 0.996 |
| Rad_Thalamus | 248 | 0.992 |
| Rad_Cerebellum | 248 | 0.992 |
| Rad_Lentiform_Nucleus | 248 | 0.992 |
| Rad_Septum_Pellucidum | 248 | 0.992 |
| Rad_Internal_Capsule | 234 | 0.936 |
| Rad_Caudate_Nucleus | 189 | 0.756 |
| Rad_Intracerebral_Haemorrhage | 158 | 0.632 |
| Rad_Insular_Cortex | 140 | 0.56 |
